# Direct Spectroscopic Quantification of the Absorption and Scattering Properties for Single Aerosol Particles

**DOI:** 10.1021/acs.jpca.2c00532

**Published:** 2022-02-23

**Authors:** Jamie W. Knight, Joanna V. Egan, Andrew J. Orr-Ewing, Michael I. Cotterell

**Affiliations:** † School of Chemistry, University of Bristol, Cantock’s Close, Bristol, U.K. BS8 1TS; ‡ School of Chemistry, University of Leeds, Woodhouse Lane, Leeds, U.K. LS2 9JT

## Abstract

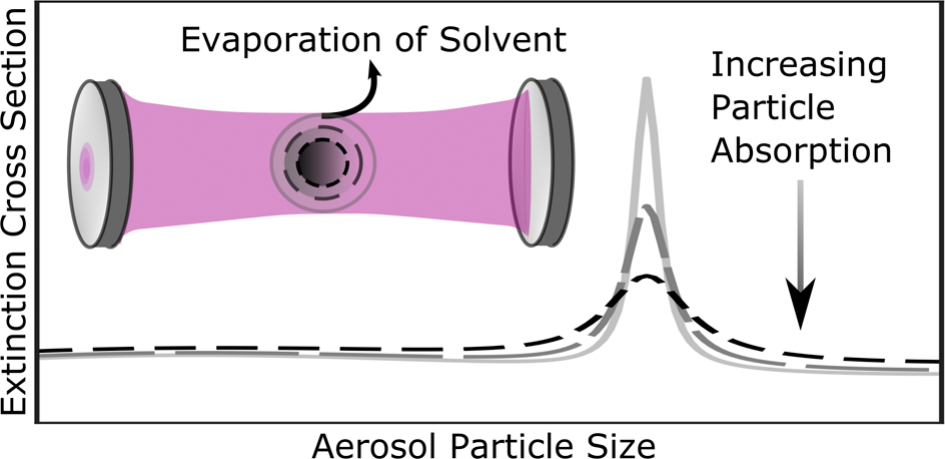

Understanding the optical properties of micrometer-scale light-absorbing aerosol particles is of paramount importance in addressing key challenges in atmospheric and physical chemistry. For example, the absorption of solar radiation by atmospheric aerosols represents one of the largest uncertainties in climate models. Moreover, reaction acceleration within the unique environments of aerosol droplets cannot be replicated in bulk solutions. The causes of these reaction rate enhancements remain controversial, but ultrasensitive spectroscopic measurements of evolving aerosol optical properties should provide new insights. We demonstrate a new approach using cavity ring-down spectroscopy that allows the first direct spectroscopic quantification of the continuously evolving absorption and scattering cross sections for single, levitated, micrometer-scale particles as their size and chromophore concentration change. For two-component droplets composed of nigrosin and 1,2,6-hexanetriol, the unprecedented sensitivity of our measurements reveals the evolving real and imaginary components of the refractive index caused by changes in concentration as 1,2,6-hexanetriol slowly evaporates.

## Introduction

Atmospheric aerosols affect climate through their interactions with solar and terrestrial radiation. However, large uncertainties persist in the magnitudes of the scattering and absorption of incident radiation by these atmospheric particles, particularly for aerosols such as brown carbon (BrC) particulates composed of organic species that absorb solar radiation at visible wavelengths.^1^ The complex refractive index, *m* = *n* + i*k*, quantifies the intensive optical properties of the material constituting an aerosol particle, with the real component *n* depending on the mean molecular polarizability and density of the particle and the imaginary component *k* characterizing the attenuation (absorption) of light. Accurate and sensitive measurements of the complex refractive indices for absorbing aerosol species are urgently needed to improve the current descriptions of the impact of aerosols in atmospheric and climate models.^1−3^ Such measurements should also be able to explore the complex atmospheric aging of aerosols, which will be influenced by multiphase chemical processes, photoinitiated chemical reactions within the particles, and gas-particle partitioning of semivolatile species. Moreover, spectroscopic monitoring of *m* could provide critical insights into how the unique physicochemical properties of aerosol particles affect reaction rates,^4^ including attributions of accelerated rates to specific properties of the particle and its ambient environment.^5,6^

**Figure 1:**
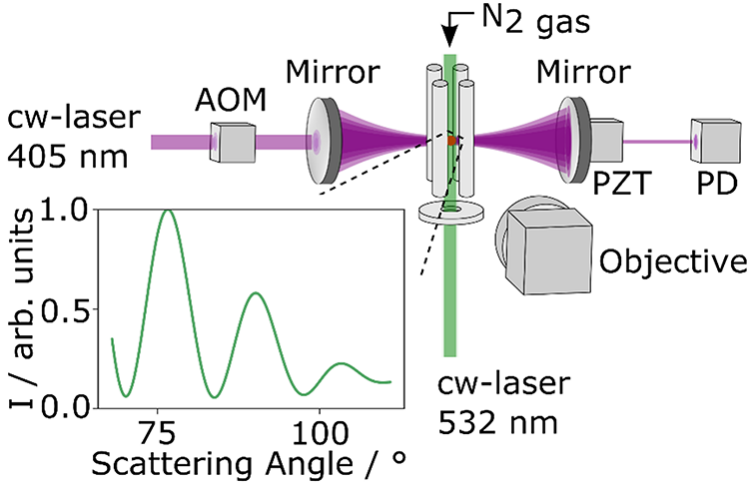
Schematic diagram of the single-particle CRDS approach for spectroscopic interrogation at a wavelength of 405 nm for single aerosol particles levitated in an LEQ trap, with angularly resolved elastic light scattering from the 532 nm laser to retrieve the particle size. AOM is an acousto-optic modulator, PZT is a piezo-electric actuator, PD is a photodiode, and I is the elastic light scattering intensity.

Single aerosol particle spectroscopy offers greater accuracy and precision in the determination of complex refractive indices than measurements on ensembles of particles.^7−9^ Combining this single-particle spectroscopy with particle trapping and levitation then enables the study of processes occurring on time scales relevant to atmospheric aerosols, which can have lifetimes of several days. Such extended measurements are beyond typical atmospheric chamber approaches. Photoacoustic spectroscopy has recently been combined with optical trapping techniques to measure light absorption properties for single aerosol particles^10^ but can suffer from artifacts arising from latent heat energy transfer pathways that confound data interpretation when particles contain volatile species.^11,12^ Price et al. instead used a linear electrodynamic quadrupole trap to confine single droplets and then recorded spectra of the elastically scattered light from an incident broadband light-emitting diode light source.^13^ Their approach potentially provides complex refractive indices for light-absorbing aerosol droplets across a range of wavelengths, although the accuracy and precision of these retrievals are not yet clear for light-absorbing droplets. Similarly, Bluvshtein et al. used photophoretic spectroscopy to probe single particles with weak absorption strengths levitated in a double-ring electrodynamic balance, but the extension of these measurements to particles with absorption strengths and particle sizes relevant to many aerosol science applications is uncertain.^15^ The approach we adopt uses cavity ring-down spectroscopy (CRDS) to measure the aerosol extinction cross section (σ_ext_), total power scattered and absorbed from an incident light beam, directly and with high sensitivity owing to the several-kilometer path length of the probe beam inside an optical cavity.^16^ Our CRDS measurements have previously provided σ_ext_ values for single nonabsorbing aerosol particles levitated in optical traps,^17−19^ but extending our studies to light-absorbing aerosols, as reported here, is a significant advance. Established optical trapping techniques are unable to confine absorbing particles spatially with the stability required for CRDS measurements because photophoretic forces displace particles away from regions of high light intensity.^20^ Instead, we have recently introduced a linear electrodynamic quadrupole (LEQ) trap for the stable levitation of scattering aerosols within a CRDS spectrometer,^14^ as depicted in Figure 1. The LEQ provides controlled particle levitation irrespective of absorption strength and, in combination with CRDS, enables us to characterize the optical properties of single light-absorbing particles as small as ∼1 μm in diameter.

Herein, we demonstrate the use of an LEQ trap to levitate single absorbing particles and manipulate them into the center of a CRDS probe laser beam. Extinction cross sections, complex refractive indices, and evolving droplet diameters are precisely determined from concurrent CRDS and elastic light scattering measurements for confined single particles with a range of absorption strengths. The retrieved *n* and *k* values agree well with predictions from physically based mixing rule models.

## Experimental Methods

The experimental setup (Figure 1) is based on our single-particle cw-CRDS apparatus reported previously, but with an upgrade to the cw laser system for enhanced experimental performance.^14,21^ The beam from a 405 nm laser (Toptica Photonics, TopMode 50 mW) is passed through an acousto-optic modulator, with the first-order diffraction beam coupled to a resonant mode of a high-finesse optical cavity formed by two high-reflectivity mirrors. One of these mirrors is mounted on a piezo-electric actuator which modulates the mirror position to tune the cavity into resonance with the narrow-bandwidth (<5 MHz) laser light. On resonance, the intracavity light intensity increases and is monitored by a photodiode that detects the light transmitted through the cavity. Once a threshold photodiode voltage is reached, an electrical pulse is sent to the acousto-optic modulator to extinguish the first-order diffraction beam and initiate a ring-down event. The intensity of the intracavity beam subsequently decreases exponentially. The characteristic time taken for the light intensity to decrease by a factor of 1/*e* is known as the ring-down time, τ.

In the experimental measurements reported here, aqueous solutions containing 1,2,6-hexanetriol (HT; Sigma-Aldrich; 96%) or mixtures of HT with water-soluble nigrosin dye (Sigma-Aldrich, CAS code: 8005-03-6, batch number BCBG2998 V) were loaded into a droplet-on-demand dispenser placed close to an induction electrode. The induction electrode imparted an ion imbalance as ∼20 μm diameter droplets were dispensed and injected into the LEQ trapping cell. N_2_ gas flowing downward through the LEQ trapping cell maintained a low relative humidity (<10%), and the water in the trapped droplets evaporated rapidly (within ∼1 s) to equilibrate with this low-RH environment, leaving a droplet with a radius of ∼1600 nm. An additional flow of dry HEPA-filtered N_2_ passed over the faces of the cavity mirrors and along the length of the optical cavity toward the CRDS entrance apertures of the LEQ trapping cell, thereby purging the cavity of dust and light-absorbing gas species that could confound our extinction measurements on single particles. The cavity was otherwise sealed from the ambient laboratory environment. Although a capability that was not exploited in the current work, this design allows the properties of the droplet’s surroundings such as relative humidity to be controlled. AC voltages were applied to pairs of diametrically opposite rods of the LEQ trap, confining the particles in the horizontal plane. A voltage applied to a bottom DC electrode exerted a repulsive electrostatic force that countered the droplet weight and the drag force exerted by the N_2_ gas flow. Constant feedback, based on measurements of the particle position using an imaging camera, adjusted this voltage to maintain the droplet position at a fixed height and at the center of the intracavity TEM_00_ mode.

Measurements of the ring-down times for both the empty cavity (τ_0_) and for the particle levitated at the center of the cavity TEM_00_ mode (τ) were recorded during the evaporative loss of semivolatile HT, with typical measurements lasting ∼2 h. The difference in the reciprocals of these ring-down times is proportional to σ_ext_:
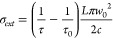
1In eq 1, *L* is the cavity length, *c* is the speed of light, and *w*_0_ is the beam waist of the TEM_00_ Gaussian cavity mode at the location of the trapped particle. Simultaneously, the particle radius was monitored using elastic light scattering. Concurrent with the CRDS measurements, the angular dependence to the elastically scattered light intensity for the levitated particle irradiated with a 532 nm laser beam was collected with a high numerical aperture (0.42) objective and imaged using a CMOS camera. These elastic light scattering measurements were recorded over the scattering angular range of ∼67.5–112.5°. The particle size was determined from comparisons of these light scattering distributions with Mie theory predictions. In these comparisons, we assumed that *k*_532_ = 0 (with the subscript “532” indicating the optical wavelength in nm) for all droplets presented in this manuscript, while *n*_532_ was optimized assuming a constant refractive index for the evolving droplet composition using a retrieval approach described in our previous publications.^21^ We recognize that these assumptions are contrary to knowledge that nigrosin scatters and absorbs strongly across the visible spectrum. However, we will show in a future publication that these assumptions lead to biases in the retrieved particle radius of only a few nanometers for our weakly light absorbing droplets for which the true *n*_532_ and *k*_532_ parameters are expected to reach values of up to 1.50 and 0.02, respectively. Moreover, the excellent correspondence between measured and simulated peak positions in the extinction cross section demonstrated here and the requirement for small corrections to the retrieved radii of no more than 5.4 nm (see below) further vindicates our particle size analysis approach.

Our approach is currently limited to particles with radii in the range of ∼500–1500 nm. The smaller size is determined by the Rayleigh limit of charge; when this limit is reached, the instantaneous expulsion of mass and charge occurs from the particle to the extent that an isolated droplet is no longer held in the LEQ trap. In principle, this lower size limit can be further reduced by lowering the charge imparted to the initial droplet at the droplet-on-demand dispenser. Although our LEQ can isolate very large (>20 μm radii) particles, the current upper size limit of our CRDS-LEQ approach is a consequence of the degraded precision of the recorded ring-down times at larger particle sizes. We hypothesize that the cause is the greater amplitude of particle motion within the AC potential of the linear electrodynamic quadrupoles; an initial inspection of in-focus images of our droplets suggests that this driven harmonic motion is larger for particles with radii of ∼1500 nm than for those with radii of <1000 nm. This greater displacement amplitude from the trap center moves the particle orthogonally to the axis of the TEM_00_ cavity mode such that the ring-down time is biased to higher values than those expected for the TEM_00_-centered particle, creating an exaggerated envelope of ring-down times at a larger particle size. In our prior experimental measurements using a Bessel beam optical trap, a smaller envelope of ring-down times was observed at comparable particle radii, suggesting that the Bessel beam optical trap provided tighter droplet confinement in the transverse directions than our current LEQ apparatus. The differences reflect the driven harmonic motion of a particle within the electrodynamic trap, in contrast to passive and stochastic (i.e., Brownian) particle motion within the optical potential of a Bessel laser beam trap. Our initial analysis indicates that in our current LEQ trap, ring-down time measurements of trapped droplets with radii in the range of 1000–1500 nm have a standard deviation higher by up to a factor of 2 than our prior measurements with a Bessel beam trap. Further optimization of our LEQ trap design to improve the stiffness of the trapping potential should remedy this shortcoming of our current apparatus.

## Results and Discussion

**Figure 2:**
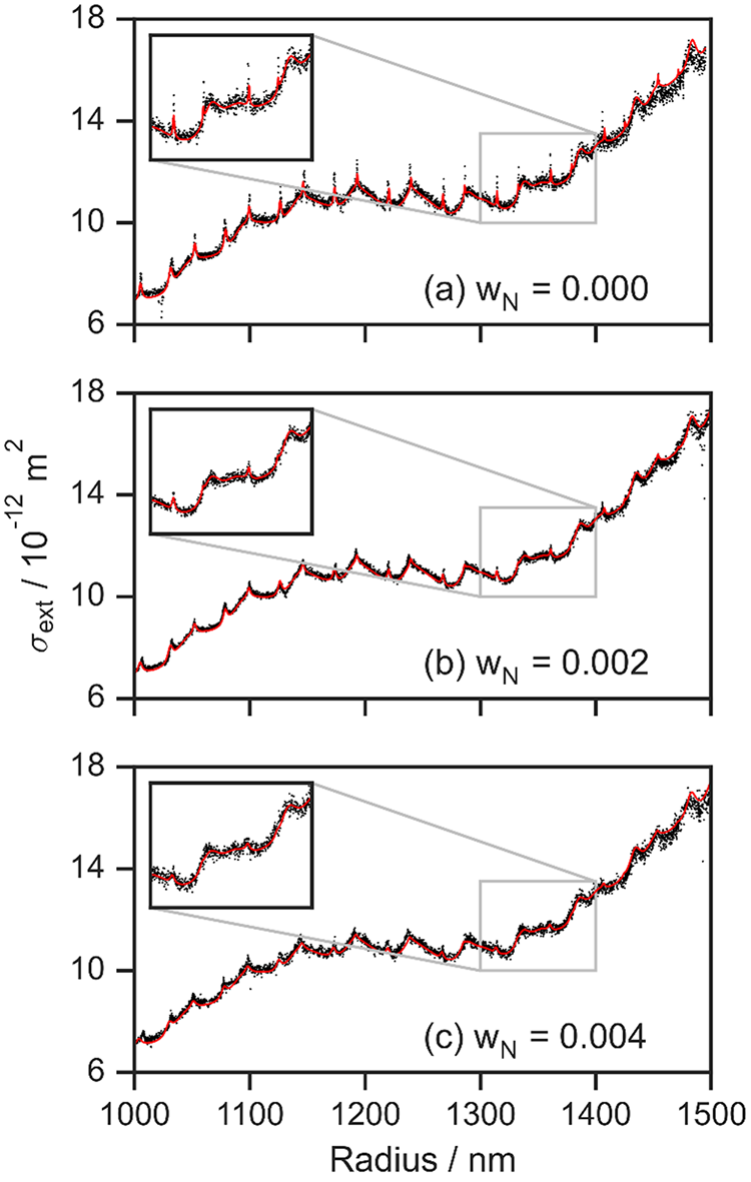
Measured (black points) variation in σ_ext_ with the particle radius averaged to a 1 Hz sampling rate for (a) a nonabsorbing HT droplet and for absorbing HT-nigrosin droplets with (b) *w*_N_ = 0.002 and (c) *w*_N_ = 0.004. The best-fit Mie theory distributions are overlaid (red lines). Insets show expanded regions of the plots to highlight changes in the whispering gallery mode structure with increasing nigrosin absorption.

Figure 2 compares the measured variations in σ_ext_ with particle radius for a droplet composed only of nonabsorbing (*k* = 0 at visible wavelengths) semivolatile HT and for two-component droplets composed of mixtures of HT with an absorbing nonvolatile dye, nigrosin. At λ = 405 nm, *k* ≈ 0.16 for the nigrosin,^7^ and the initial nigrosin mass fraction (*w*_N_) for the binary HT-nigrosin droplets was 0.002 or 0.004. The particle size decreased over time as the semivolatile HT evaporated, while the concentration of the nonvolatile nigrosin increased in the two-component droplets, thereby increasing the imaginary refractive index of the droplet at smaller radii. The size-dependent σ_ext_ data reveal broad oscillatory structure caused by the interference of the light rays transmitted through and diffracted around the droplet, with superimposed sharp resonance structures caused by the efficient coupling of the light into resonant modes (referred to as whispering gallery modes) of the spherical particle.^22^ The remarkable sensitivity of our measurements is evident from the distinguishable impacts of only small increases in the initial chromophore mass concentration corresponding to 0.2% of the total droplet mass. The whispering gallery modes decrease in amplitude and broaden as the nigrosin concentration increases because of greater absorption losses for these resonant modes (and hence a lower quality factor of the resonant mode for the droplet) as *k* increases.^22^ Therefore, the resolved resonance structure in the extinction measurements contains valuable information regarding the absorption efficiency of weakly absorbing aerosol particles and may be compared to optical models to retrieve the complex refractive index.

Previous publications have presented the application of cavity standing-wave Mie theory to retrieve the real refractive index for scattering particles, accounting for the standing wave formed inside a linear optical cavity.^18,21,23−25^ An envelope of measured σ_ext_ values results from the Brownian motion of the particle sampling different phases of this standing wave, with the limits of the envelope corresponding to the particle centered at a node or an antinode. As we will show in a forthcoming publication, the measured σ_ext_ values (recorded at a sampling rate of ∼20 Hz) converge to the Mie theory limit for a plane wave when averaged to a sampling rate of 1 Hz, and we exploit this method of data analysis here to improve the accuracy and precision of complex refractive index retrievals. The description of the complex refractive index is optimized by performing a least-squares fit of Mie theory to the measured 1 Hz cross sections by minimizing the merit function (χ):
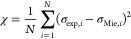
2In eq 2, *N* is the number of data points, σ_exp,*i*_ is the measured extinction cross section at a given particle radius, and σ_Mie,*i*_ is the corresponding calculated Mie theory cross section at the same particle radius. The σ_exp,*i*_ value in this expression is the mean value calculated over a 1 s interval; therefore, both σ_exp,*i*_ and the particle radius (which is used in the calculation of σ_Mie,*i*_) are recorded at a sampling rate of 1 Hz. Our method fits the magnitudes of the full σ_exp,*i*_ data set at all particle radii, not just the positions and widths of the resonant modes, so it is not limited by the broadening of these modes with increasing *k*. Importantly, because the concentrations of HT (volatile) and nigrosin (nonvolatile) change with the evaporation of the HT component, refractive indices *n* and *k* depend on the particle size. The size-dependent real refractive index can be described by the following empirical expression:
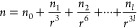
3In eq 3, *r* is the droplet radius, *n*_0_ is the real refractive index of the droplet in the limit of infinite size (corresponding here to the *n* for pure HT), and *n*_*l*_ represents the coefficients characterizing the size dependence of *n* for the droplet.^21^ For our measurements, only *n*_1_ needs to be varied in this expression to reconcile our Mie theory model calculations with our measured dependence of σ_exp_ on particle size, with all other *n*_*l*_ values set to zero. The size-dependent imaginary refractive index is defined by
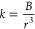
4In eq 4, *B* is a constant. This expression has a general form consistent with the mass fraction mixing rule (Supporting Information). Parameters *n*_1_, *B*, and *w*_0_ and a small multiplicative factor applied to the retrieved particle radius (equivalent to absolute changes in particle radii of less than 5.4 nm) are optimized using a grid search algorithm to minimize the value of χ. Figure 2 compares the best-fit Mie theory distributions to our measurements. Corresponding contour plots for the variation in χ with *n*_1_ and *B*, for optimized values of *w*_0_ and the radius correction factor, are presented in the Supporting Information and demonstrate a single minimum.

**Figure 3:**
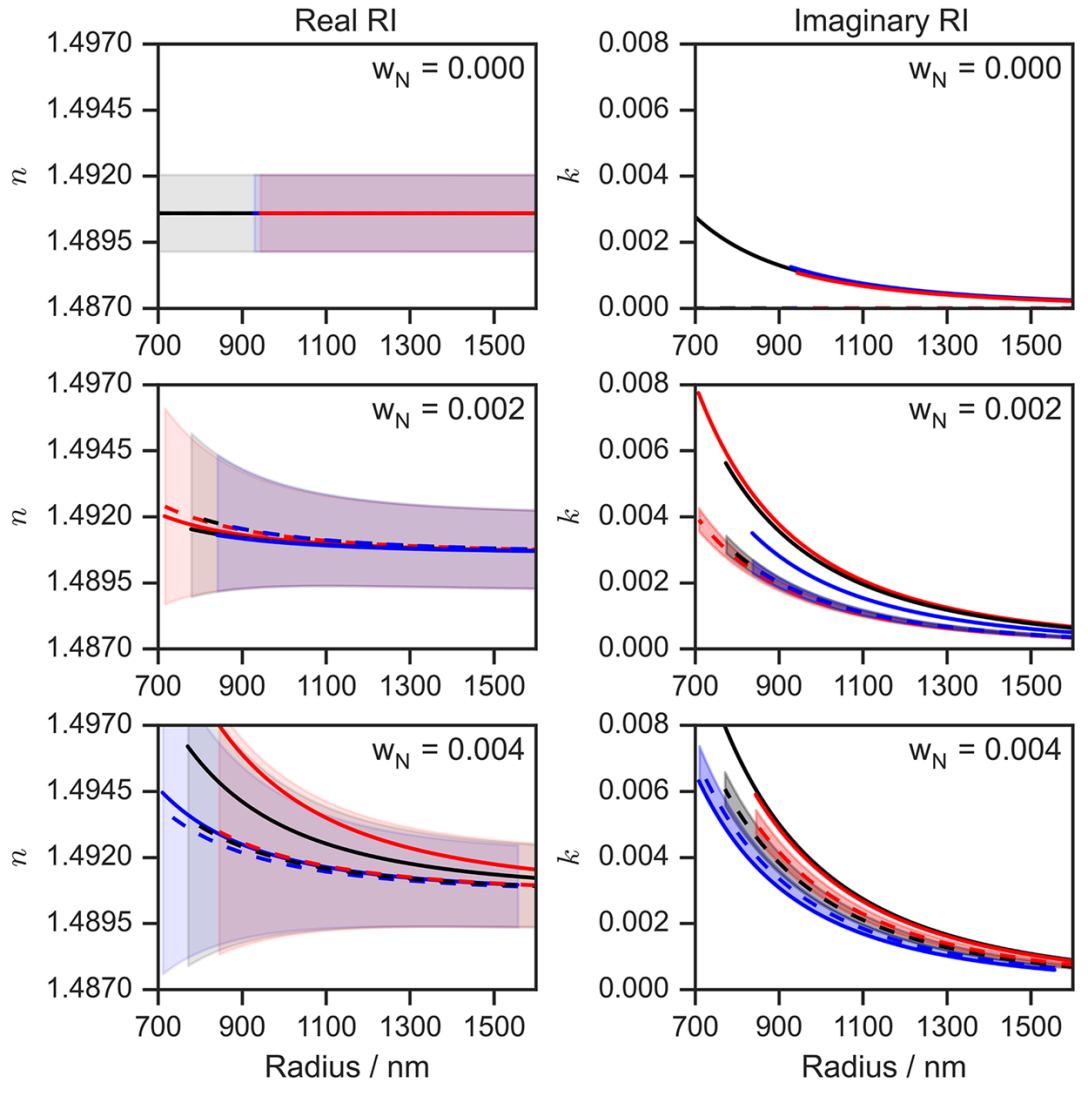
Retrieved (solid lines) and predicted (dashed lines) variations of the *n* and *k* components of the complex refractive indices with particle radius for pure HT droplets (*w*_N_ = 0.000; top) and for HT-nigrosin droplets with initial *w*_N_ values (corresponding to the solution composition loaded into our droplet dispensers) of 0.002 (middle) and 0.004 (bottom). The shaded envelopes represent the uncertainties in the predicted *n* and *k* values as a function of particle size. The three solid lines of different colors in each panel correspond to the retrieved refractive indices for three separate repeat droplets that were studied for each initial droplet *w*_N_ value. The data series extends to different lower values for droplet radii because droplets fell out of the trap stochastically upon evaporation to radii of <900 nm.

For each nigrosin mass fraction (0.000, 0.002, and 0.004), repeat measurements were performed on three separate droplets, with the measured variations in σ_ext_ with particle size provided in the Supporting Information. Figure 3 compares the retrieved size-dependent refractive indices for each droplet studied with predictions from physically based refractive index mixing rule models.

The mixing rule models used in this work are described in the Supporting Information. The predictions of *n* and *k* depend on the initial size of the HT-nigrosin droplets, which could vary from one droplet to the next for sequentially generated droplets. This variability in initial droplet size introduces differences in the size-dependent refractive indices, for the predicted as well as the retrieved values. Therefore, Figure 3 shows the size-dependent refractive index predicted for each individual droplet (as indicated by the color of the data series) using the best estimate of the initial droplet size as determined from angularly resolved elastic light scattering measurements.

The analysis for the pure HT droplets retrieved nonzero *B* values, corresponding to *k* values in the range of 0.000–0.003 at the largest and smallest particle sizes, respectively. We do not suggest from this result that HT is absorbing. Instead, this nonzero value arises because the retrievals of *k* are sensitive to small errors (of less than 5 nm) in the determined droplet radii that are typical of our experimental approach, causing a small misalignment of the sharp resonance structure in the measured and calculated σ_ext_ values. A comprehensive assessment of the impact of particle sizing uncertainties on the fidelity of complex refractive index retrievals is ongoing. This assessment involves the simulation of artificial measurement data sets of ring-down times and particle sizes for evaporating particles with varying absorption strengths (*k*), with representative measurement uncertainties (including random uncertainties and systematic biases) added to these synthetic data sets prior to the retrieval of *n* and *k* using the methods described here. In principle, there is no upper limit to our retrieved *k* values from the measurements of σ_ext_ and particle size. The least-squares fit of Mie theory to our σ_ext_ measurements incorporates both the sharper resonance structure and the broader underlying interference structure which become progressively damped as *k* increases. This comprehensive fitting approach is evident from the agreement between the best-fit Mie theory distributions in Figure 2, which match the measurements across the whole extinction cross-section range not just at the locations of the resonance peaks. Nonetheless, our approach is limited by the ability to retrieve particle sizes from measured phase functions.

We retrieve *n*_1_ values of less than 10^–26^ m^3^ for pure HT droplets, corresponding to a negligible perturbation of the pure component *n* for HT by <10 ppb for our droplet sizes. The retrieved *k* values show clear increases as the value of *w*_N_ for the initially dispensed solution increases from 0.000 to 0.004 in a 0.002 interval. While the precision of our technique therefore remains high for the complete range of *k* values studied in this article, the accuracy deteriorates at small *k* (*w*_N_ ≤ 0.002). This reduction in the accuracy is exaggerated by the 1/*r*^3^ factor in eq 4, with small changes in *B* introducing large differences in *k* at small particle sizes. This effect is particularly significant at smaller *w*_N_ because the retrieved *B* is close to the sensitivity limit. Nevertheless, the retrieved *n* and *k* show good reproducibility for a given initial HT-nigrosin mass fraction. The differences in these retrievals arise mostly from the variability in the initial size of the particles generated by the droplet dispenser; for a given solution concentration and voltage applied to the droplet-on-demand dispenser, there is a natural variability in the diameter of the ∼20 μm sequentially generated droplets of up to a few hundred nanometers. Sources of uncertainty in the mixing rule model predictions are discussed in the Supporting Information.

## Conclusions

Here, we have demonstrated that a combination of LEQ trapping and CRDS measurement allows direct, contact-free retrievals of both the real and imaginary components of the complex refractive index with high sensitivity for single light-absorbing droplets. The outcomes allow predictive mixing rule models to be tested for droplets with constantly evolving compositions. We emphasize the small changes in *k* of <0.002 resolved in our measurements, with light-absorbing aerosols in the atmosphere known to exhibit *k* values at visible wavelengths of >0.1 for BrC^26^ and up to 0.8 for black carbon.^27^ An alternative approach recently developed by Price et al. uses a broadband light-emitting diode (LED) source to record spectra from a droplet confined in an LEQ.^13^ While this method can potentially measure complex refractive indices for aerosol droplets across the bandwidth of the LED, the accuracy and precision of these retrievals are not yet clear for light-absorbing droplets with *k* > 10^–3^. Our method has the advantage of retrieving *k* values directly by fitting a light scattering model to recorded extinction cross-section measurements. It also successfully extracts useful optical information for the submicrometer-sized particles of most relevance to atmospheric aerosols and can be applied to particles with values of *k* (>10^–4^) that are most uncertain in climate models. Bluvshtein et al. showed that photophoretic spectroscopy could probe single light-absorbing particles levitated in an electrodynamic balance to determine *k* with a sensitivity of ∼10^–5^ for droplets with *k* in the range of ∼10^–5^–10^–4^.^15^ Mie resonance spectroscopy was used to determine the particle size and real component of the refractive index for particles with radii of ∼7–13 μm. However, it is not clear that this experimental approach can be extended to particles with radii below 1 μm or to more strongly absorbing particles. Our work demonstrates retrievals of *k* as high as ∼10^–2^ for particles with radii of as small as ∼700 nm. Wider advantages of our approach include the direct measurement of extinction cross sections that are of relevance to radiative forcing calculations in climate models, for both spherical and nonspherical particles.^14^

Our combination of CRDS and LEQ trapping offers an platform for resolving an array of challenges at the forefront of aerosol science. These challenges include understanding the impact of the mixing state on light absorption, such as the role of coatings on strongly absorbing black carbon particulates;^28,29^ quantifying the impacts of particle size, viscosity, and chemical composition on photoinitiated processes such as photobleaching for which the atmospheric time scales for organic chromophores are not known accurately;^30^ and understanding the enhanced reaction rates that are increasingly being reported in aerosols, including those for the formation of chromophores relevant to atmospheric BrC aerosols.^31,32^

## Supporting Information Available

Origin of size-dependent refractive indices, details of the retrieval algorithm for processing cross section versus particle size data, comprehensive description of the mixing rule models used, choice of input values for these models, impact of uncertainties in the models on the uncertainties in predicted and retrieved *n* and *k*, plots of the measured variation in σ_ext_ with particle size for each droplet studied with the overlaid best-fit Mie theory distributions, and the corresponding contour plots of merit function with variations in *n*_1_ and *B* for these droplets (PDF)
