# Supplementary material for: Direct Spectroscopic Quantification of the Absorption and Scattering Properties for Single Aerosol Particles
Source: J Phys Chem A. 2022 Feb 23;126(9):1571–7. doi: 10.1021/acs.jpca.2c00532 (PMC9097522; doi:10.1021/acs.jpca.2c00532)
Supplement: Supplementary file 1 — jp2c00532_si_001.pdf [file jp2c00532_si_001.pdf]

Supporting Information:

Direct Spectroscopic Quantification of the Absorption and Scattering Properties for Single Aerosol Particles

Jamie W. Knight,[1] Joanna V. Egan,[1,2] Andrew J. Orr-Ewing,[1]* Michael I. Cotterell[1]*

[1]School of Chemistry, University of Bristol, Cantock's Close, Bristol, UK, BS8 1TS

[2]School of Chemistry, University of Leeds, Woodhouse Lane, Leeds, UK, LS2 9JT

**Correspondence to*:

Michael I. Cotterell (m.cotterell@bristol.ac.uk)

Andrew J. Orr-Ewing (a.orr-ewing@bristol.ac.uk)

[S1] The size dependent complex refractive index

Two-component droplets of 1,2,6-hexanetriol (HT) and nigrosin were generated with initial mass fractions of nigrosin of 0.002 and 0.004. At low RH (<10 %) with N_2 gas flowing over the particle, the semi-volatile 1,2,6-hexanetriol (HT) evaporated leaving an increasing mass fraction of the non-volatile nigrosin. Therefore, to describe the evolving droplet optical properties, size dependent refractive indices are defined.

The imaginary refractive index (Eqn. (4) of the main text) is defined as:

$$k = \frac{B}{r^3} \qquad \text{(S1)}$$

In Eqn. (S1), r is droplet radius and B is a constant. Eqn (S1) has a physical basis and may be derived from the mass fraction (w) mixing rule. The mass fraction mixing rule, defined below, is consistent with our understanding of how k relates to bulk light absorption coefficients, which depend linearly on the concentration of absorbing chromophores through the Beer-Lambert law. We therefore define k as a linear mass fraction weighting of the k_i for the individual components:

$$\begin{aligned} k = \; & k_{\mathrm{HT}} \mathrm{w}_{\mathrm{HT}} + \; k_{\mathrm{N}} \mathrm{w}_{\mathrm{N}} \\ & = k_{\mathrm{HT}}(1 - \mathrm{w}_{\mathrm{N}}) + \; k_{\mathrm{N}} \mathrm{w}_{\mathrm{N}} \\ & = k_{\mathrm{HT}} + \mathrm{w}_{\mathrm{N}}(k_{\mathrm{N}} - k_{\mathrm{HT}}) \end{aligned}$$

in which the subscripts 'HT' and 'N' denote terms corresponding to 1,2,6-hexanetriol and nigrosin, respectively. We can express the mass fraction of N in terms of the absolute mass of N in the droplet (m_{N}) and the total droplet mass (m_{T}), $\mathrm{w}_{\mathrm{N}} = m_{\mathrm{N}}/m_{\mathrm{T}}$. Moreover, we can write m_{T} as the product of the particle volume (V) and particle effective density (ρ_{e}). Therefore, we write the k of the droplet as:

$$k = \; k_{\mathrm{HT}} + \frac{m_{\mathrm{N}}}{V \rho_e}(k_{\mathrm{N}} - k_{\mathrm{HT}})$$

For our spherical droplets with a radius r, the effective k of the droplet can be described in terms of particle size using:

$$k = \; k_{\mathrm{HT}} + \frac{3 m_{\mathrm{N}}}{4 \pi r^3 \rho_e}(k_{\mathrm{N}} - k_{\mathrm{HT}})$$

In our single particle measurements, the particle size evolves continuously due to the evaporation of HT, but the terms k_{N}, k_{HT}, and m_{N} are constant. Moreover, assuming ideal mixing, the maximum change in ρ_{e} during an experiment is predicted to be <0.02 g cm^{-3} for a ρ_{e} value of ~1.10 g cm^{-3} and thus can be assumed invariant with particle size. Therefore, we can combine the terms $3 m_{\mathrm{N}}(k_{\mathrm{N}} - k_{\mathrm{HT}})/(4 \pi \rho_{\mathrm{e}})$ into a single term that we denote B. The term B is constant for a given droplet, but can vary from one droplet to another owing to changes in the component mass fractions. Therefore, we write k as:

$$k = \; k_{\mathrm{HT}} + \frac{B}{r^3}$$

In the limit of infinite size, the droplet consists of pure HT, and $k = k_{\mathrm{HT}} = 0$. Therefore, the size dependent imaginary refractive index may be expressed as Eqn (S1).

The size dependent real refractive index is defined by the empirical expression:

$$n = n_0 + \frac{n_1}{r^3} + \frac{n_2}{r^6} + \cdots + \frac{n_l}{r^{3l}} \qquad \text{(S2)}$$

In Eqn (S2), n_0 is the real refractive index of the droplet in the limit of infinite size (corresponding here to the n for pure HT), and n_l are coefficients characterizing the size dependence of n for the droplet.[1]

[S2] Least-squares fitting procedure

The light field inside the optical cavity used for our ring-down experiments is a standing wave. A spherical particle trapped inside the optical cavity may be centred on a node, anti-node, or any phase between these two limits of the field. The magnitude of the light extinction by the particle depends on its position within this field, with the particle continuously undergoing Brownian motion over length scales greater than the distance between the positions of these nodes and anti-nodes. Previous publications by our group used cavity standing wave generalized Lorentz-Mie Theory (CSW-GLMT) to predict extinction cross sections (σ_{ext}) at any arbitrary location within the standing wave field. Comparisons of the measured cross sections to CSW-GLMT allow retrievals of the complex refractive index.

In this manuscript, we propose a new method to retrieve the complex refractive index and will demonstrate in a future publication the improvements to the accuracy in these retrievals over a range of particle absorption strengths. As described in the main manuscript, measured σ_{ext} values recorded at a sampling rate of ~20 Hz can be averaged to a 1 Hz sampling rate. The cross sections then converge to the Mie theory limit for a plane wave, thus accounting for the standing wave field within the cavity by averaging over multiple particle positions sampled by Brownian motion. The result of a least-squares fit of the measured 1-Hz cross sections to Mie theory allows retrieval of the optimized complex refractive index of the droplet, by minimizing the merit function (χ):

$$\chi = \frac{1}{N}\sum_{i=1}^{N}(\sigma_{exp,i} - \sigma_{Mie,i})^2 \qquad \text{(S3)}$$

In Eqn (S3), N is the number of data points, $\sigma_{\mathrm{exp,i}}$ is the measured extinction cross section at a given particle radius, and $\sigma_{\mathrm{Mie,i}}$ is the corresponding calculated Mie theory cross section at the same particle radius.

In principle, the least-squares fit can be performed by a straightforward optimisation of n_1 and B. In practice, the value of the beam waist of the TEM_{00} Gaussian cavity mode at the location of the trapped particle (w_0) is varied around its predicted value for our optical cavity geometry to account for shifts in the particle position from the longitudinal centre of the intracavity beam. In addition, a multiplicative correction factor to the particle radius data of no more than ±0.36 % (corresponding to an absolute radius correction of up to 5.4 nm for our measurement size range) is also included to account for any small systematic bias in the retrieved radius from recorded phase functions (angularly scattered light). Therefore, the parameters n_1, B, w_0 and a radius correction factor are optimised using a grid search algorithm to minimise the value of χ.

[S3] Mixing rule models

The variation in n with particle size is predicted using a mole fraction (x) weighting of molar refraction (R):

$$R_{\mathrm{mix}} = x_{\mathrm{HT}} R_{\mathrm{HT}} + x_{\mathrm{N}} R_{\mathrm{N}} \qquad \text{(S4)}$$

In Eqn. (S4), R_{mix} is the molar refraction of the internally mixed droplet and R is defined by:

$$R = \left(\frac{n^2 - 1}{n^2 + 2}\right)\frac{M}{\rho} \qquad (S5)$$

In Eqn. (S5), M is the molecular weight.[2] This mixing rule is consistent with the Lorentz-Lorenz model describing the manifestation of the refractive index (a macroscopic quantity) from an atomistic description of matter. The pure component molar refractions (R_{HT} and R_{N}) are calculated straightforwardly from their known n, M and ρ; Sect. S4 describes how we assign values to these parameters. The values for component mole fractions (x_{HT} and x_{N}) are calculated from the known (measured) component mass fractions for the solution loaded into the droplet-on-demand dispenser, the molecular weights for the two components, and using the measured particle size and its evolution to calculate the mass loss of HT from droplets during experiments (assuming that changes in droplet size result from loss of HT only). To convert the calculated

R_{mix} from Eqn. S4 to n_{mix}, we need to know how the mean molecular weight (M_{mix}) and effective density (ρ_e) vary for the two-component, internally mixed droplet. The molecular weight is straightforward to calculate and is given by:

$$M_{\text{mix}} = x_{\text{N}} M_{\text{N}} + (1 - x_{\text{N}}) M_{\text{HT}} \qquad \text{(S6)}$$

We use the ideal mixing rule to describe ρ_e in terms of the mass fractions of HT and nigrosin:

$$\rho_e = \frac{1}{\left(\frac{w_{\text{HT}}}{\rho_{\text{HT}}} + \frac{w_{\text{N}}}{\rho_{\text{N}}}\right)} \qquad \text{(S7)}$$

In Eqn. (S7), w is the mass fraction, with w_{HT} and w_{N} calculated from the evolving mole fractions (x_{HT} and x_N) described above and the molecular weights for the two components. Eqn. (S7) assumes that the volume of mixing is zero and therefore that there are no non-ideal interactions between HT and nigrosin. We are not aware of any reports in the literature of the density for HT-nigrosin mixtures and are therefore unable to assess the validity of this expression over the complete range of mixing. However, we note that the nigrosin mole fraction in our experimental droplets does not exceed 0.01 (with the highest nigrosin mole fractions occurring at the smallest particle size for a given droplet). Therefore, in this dilute limit, we expect deviations from non-ideal mixing to be minor.

The variation in k with particle size is predicted using a mass fraction weighting of the pure component imaginary refractive indices:

$$k = k_{\text{HT}} w_{\text{HT}} + k_{\text{N}} w_{\text{N}} \qquad (S8)$$

Pure HT is non-absorbing and was assigned a value $k_{HT} = 0$, while we used $k_N = 0.163$ at λ = 405 nm.[3]

[S4] Summary of physical quantities used in the manuscript

Table S1 below summarises the values, and their uncertainties, of input variables to our refractive index model calculations.

Symbol	Description	Value	Comments
k_{HT}	Imaginary refractive index of HT	0.000 ± 0.000	-
k_N	Imaginary refractive index of nigrosin	0.163 ±0.012	Taken from Ref.[3], which uses optical spectroscopy measurements on nigrosin aerosols.
n_{HT}	Real refractive index of HT	1.4906 ± 0.0012	Taken from Ref. [1], using our previous single particle CRDS measurements on 1,2,6-hexanetriol.
n_N	Real refractive index of nigrosin	1.609 ± 0.051	Taken from Ref.[3], which uses optical spectroscopy measurements on nigrosin aerosols.
M_{HT}	Molecular weight of HT	134.17 ± 0.00 g mol^{-1}	-
M_N	Molecular weight of nigrosin	922 ± 168 g mol^{-1}	Highly uncertain composition owing to crude manufacturing process. Available literature provides values in the range 753.98 - 1090.92 g mol^{-1}.[4, 5] We take the average, and the difference between the average and the limiting values, to represent M_N and its uncertainty respectively.
ρ_{HT}	Density of HT	1.100 ± 0.003 kg m^{-3}	Taken from Ref. [6], which measured bulk liquid densities using a glass capillary pycnometer.
ρ_N	Density of nigrosin	1.632 ± 0.016 kg m^{-3}	Taken from measurements of nigrosin density in our own group. Submitted for publication (Vokes *et al.*, *Densities of Internally Mixed Organic-Inorganic Particles from Mobility Diameter Measurements of Aerodynamically Classified Aerosols*, *Aerosol. Sci. Technol.*, submitted).

Table S1. Summary of physical quantities used to calculate the real and imaginary refractive indices for our two-component droplets using the mixing rule models described in Sect. S3.

The error envelopes in Figure 3 of the main manuscript were calculated by propagating the uncertainties, summarised in Table S1, using standard propagation of uncertainty formulae. In addition, these envelopes include the impacts of uncertainties in the measured masses of HT and nigrosin in the solution loaded into the droplet-on-demand dispenser, and therefore the contribution of these uncertainties to those in the mass fractions and mole fractions of the initial levitated droplet (i.e., the droplet immediately after generation and subsequent equilibration of the droplet water activity with the RH <10% environment). When creating the mixtures of HT-nigrosin, a combined mass of 10 g for each composition was made, with an uncertainty of ± 0.0001 g in each pure component mass. The solutions were subsequently diluted to allow droplets to be generated from the droplet-on-demand dispenser with an initial radius, after complete evaporation of the water, of ~1600 nm. An uncertainty in this initial droplet radius of ± 5 nm was calculated from the combined uncertainty of our phase function measurements of the initial particle size and the estimated time between droplet generation and the first phase function acquisition. This uncertainty in the initial particle size is also reflected in the error envelopes in Figure 3.

For our error envelope calculations for n_{mix}, we note that, while the uncertainties in ρ_{HT} and ρ_N are propagated through calculations of R_{HT} and R_N as well as those for component mass fractions and mole fractions, we set the uncertainty in the mixture density, ρ_e, to zero. When uncertainties in the ideal mixing ρ_e were calculated via propagation of uncertainty, an unrealistic uncertainty in ρ_e of up to 9.9 % was obtained, even though the mixture composition deviates only marginally from the composition of a pure-HT droplet (with nigrosin mole fractions less than 0.01 for the smallest radii droplets in our study). In part, we think this large uncertainty arises because our error propagation calculations assume that the input variables are independent. Yet, some input variables are correlated (e.g., mass fractions of the HT and nigrosin components) which would reduce the calculated uncertainty in mixture density. However, we do not know the covariance between these correlated inputs. Under the assumption that the uncertainty in ρ_e is zero, M_N represents the most significant source of uncertainty in n. Specifically, we estimate that the uncertainty in M_N introduces uncertainty in predictions of n for our droplets of ≤0.0006 at particle radii of >1600 nm, and ≥0.005 for particle radii <800 nm.

In addition to the uncertainty in k_N given by Cotterell *et al.*[3] (±0.012, see Table S1), there is evidence of differences in optical properties and chemical composition of nigrosin between batches and manufacturers.[4, 5, 7-9] These differences are difficult to quantify, so are not included in our error envelope calculations. The agreement between our retrieved and predicted *k* will be most strongly degraded by this uncertainty in the imaginary refractive index of pure nigrosin.

[S5] Size dependent extinction cross-section plots and accompanying contour plots

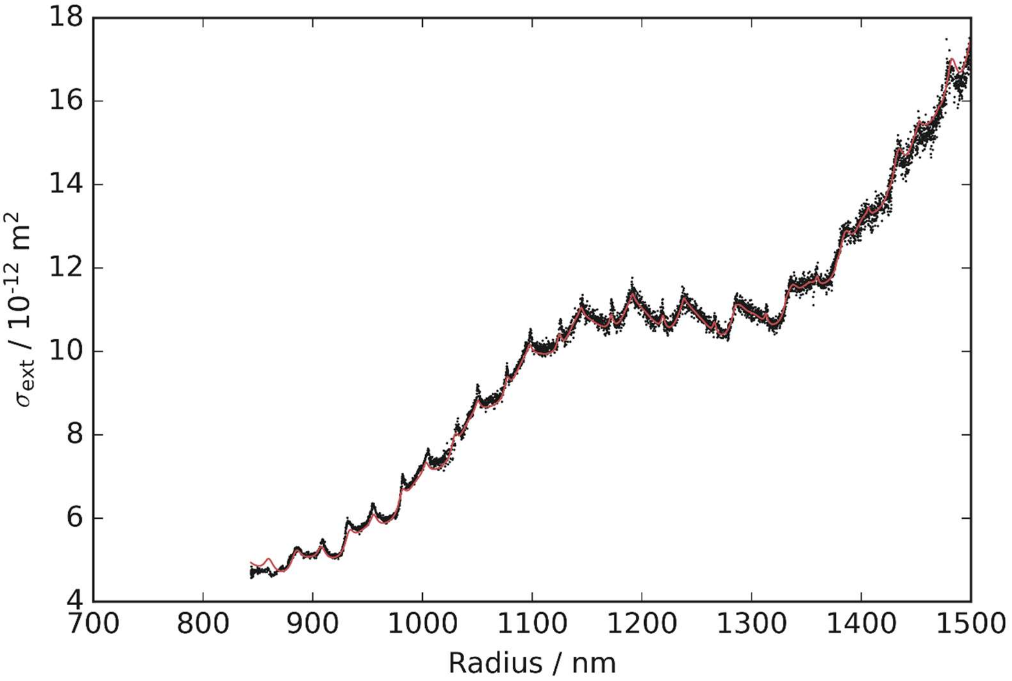

Figure S1. Measured (black points) variation in σ_{ext} with particle r for droplet 1 of 3 for the w_N = 0.004 droplets. The best-fit Mie theory distribution is overlaid (red line). The initial particle radius after complete evaporation of water was 1674 nm

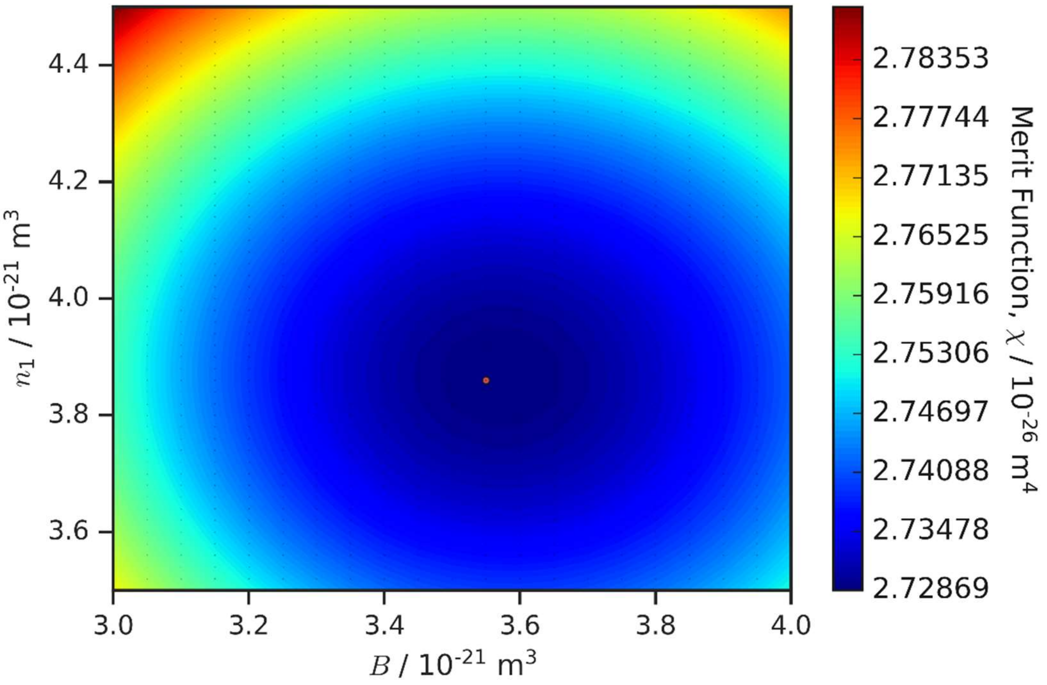

Figure S2. Contour plot depicting the calculated merit functions for droplet 1 of 3 for the w_N = 0.004 droplets, for optimized values of w_0 and the radius scale factor of 259.68 μm and 1.00171, respectively.

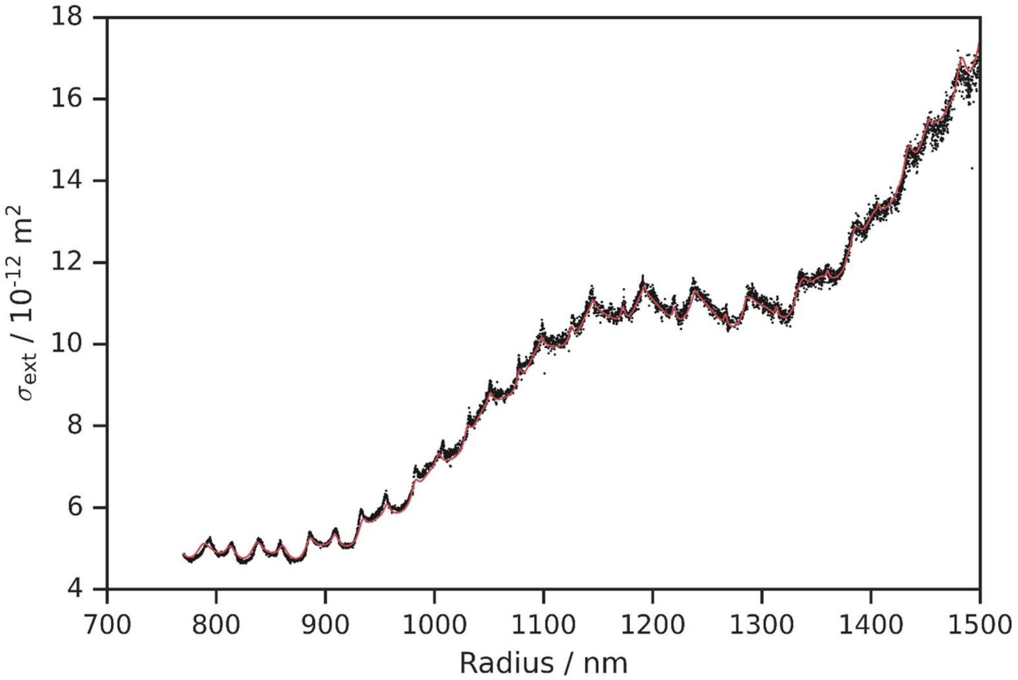

Figure S3. Measured (black points) variation in σ_{ext} with particle r for droplet 2 of 3 for the w_N = 0.004 droplets. The best-fit Mie theory distribution is overlaid (red line). The initial particle radius after complete evaporation of water was 1628 nm. This plot corresponds to the data plotted in Figure 2 of the main manuscript, for w_N = 0.004.

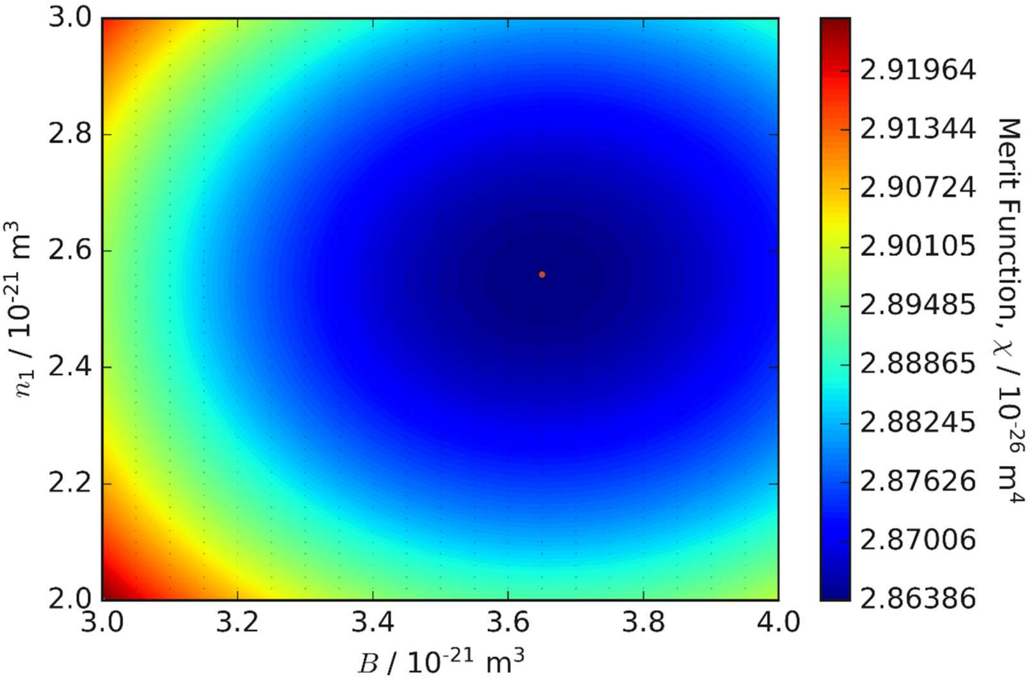

Figure S4. Contour plot depicting the calculated merit functions for droplet 2 of 3 for the w_N = 0.004 droplets, for optimized values of w_0 and the radius scale factor of 258.47 μm and 1.00352, respectively.

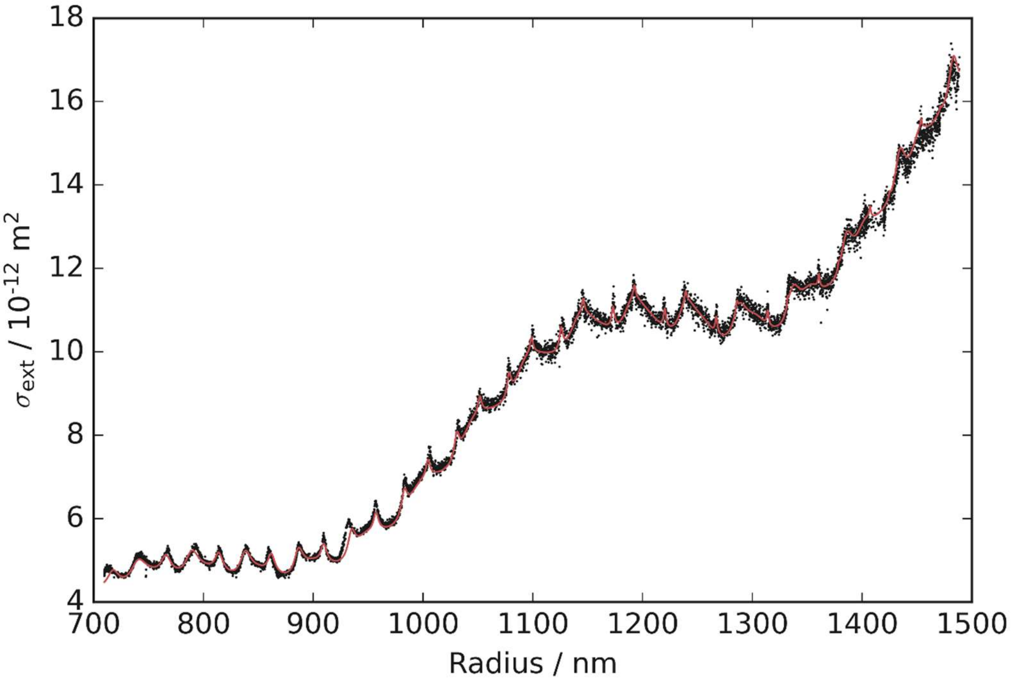

Figure S5. Measured (black points) variation in σ_{ext} with particle r for droplet 3 of 3 for the w_N = 0.004 droplets. The best-fit Mie theory distribution is overlaid (red line). The initial particle radius after complete evaporation of water was 1674 nm.

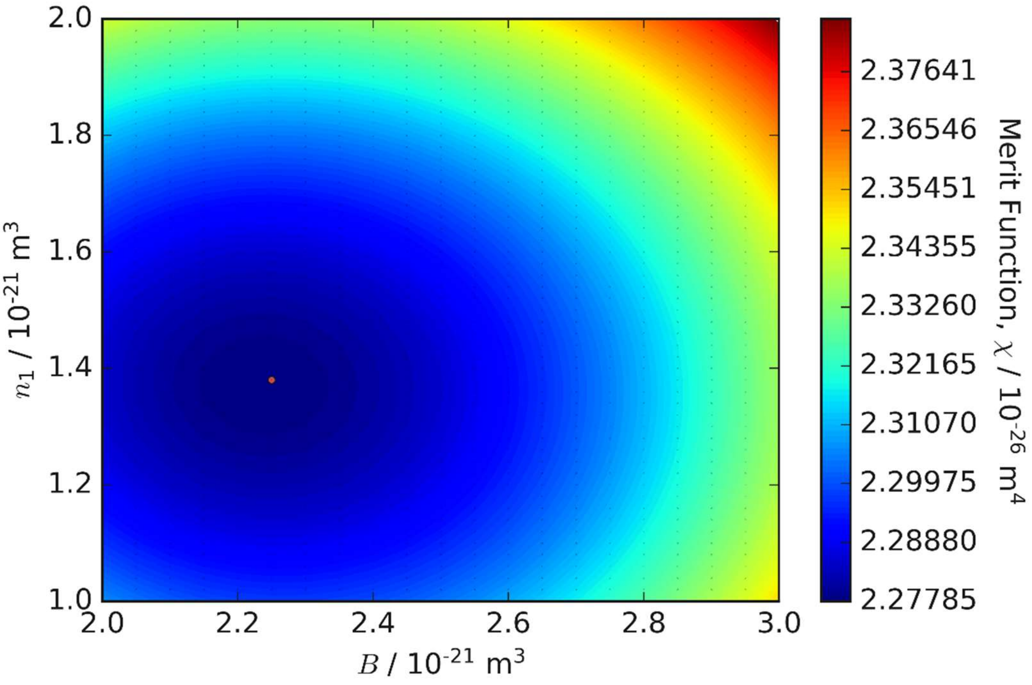

Figure S6. Contour plot depicting the calculated merit functions for droplet 3 of 3 for the w_N = 0.004 droplets, for optimized values of w_0 and the radius scale factor of 258.38 µm and 1.00222, respectively.

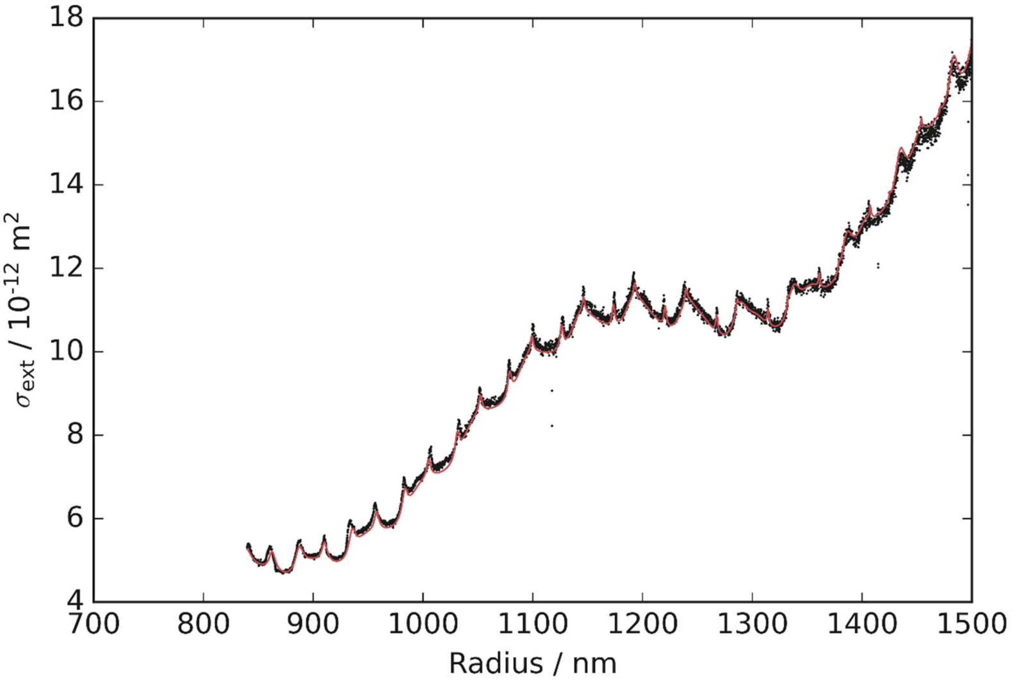

Figure S7. Measured (black points) variation in σ_{ext} with particle r for droplet 1 of 3 for the w_N = 0.002 droplets. The best-fit Mie theory distribution is overlaid (red line). The initial particle radius after complete evaporation of water was 1650 nm.

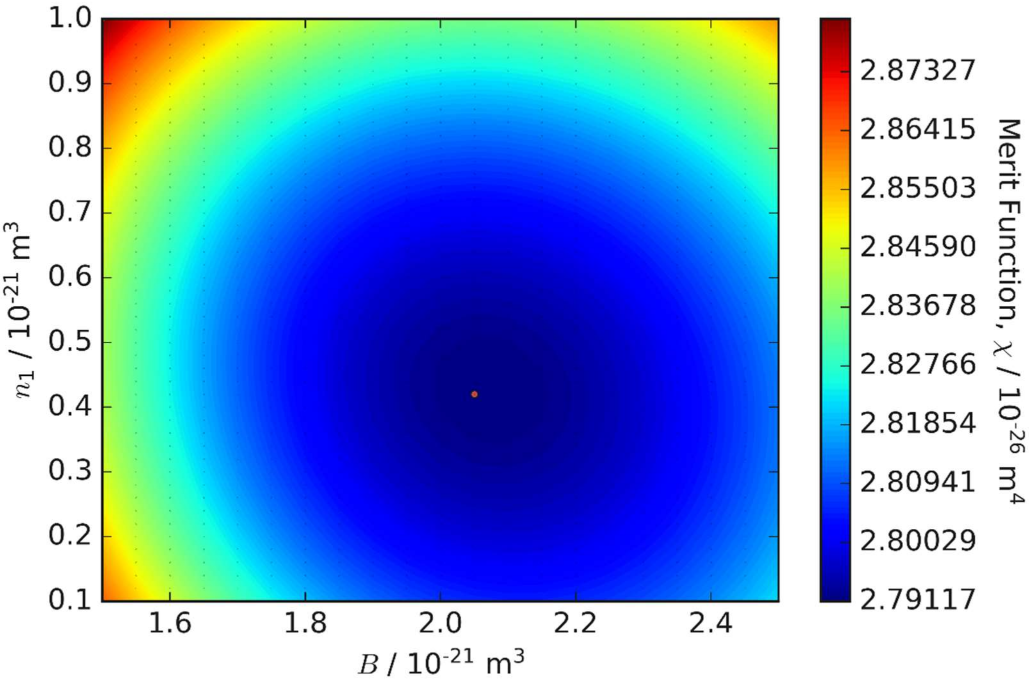

Figure S8. Contour plot depicting the calculated merit functions for droplet 1 of 3 for the w_N = 0.002 droplets, for optimized values of w_0 and the radius scale factor of 256.91 µm and 1.0017, respectively.

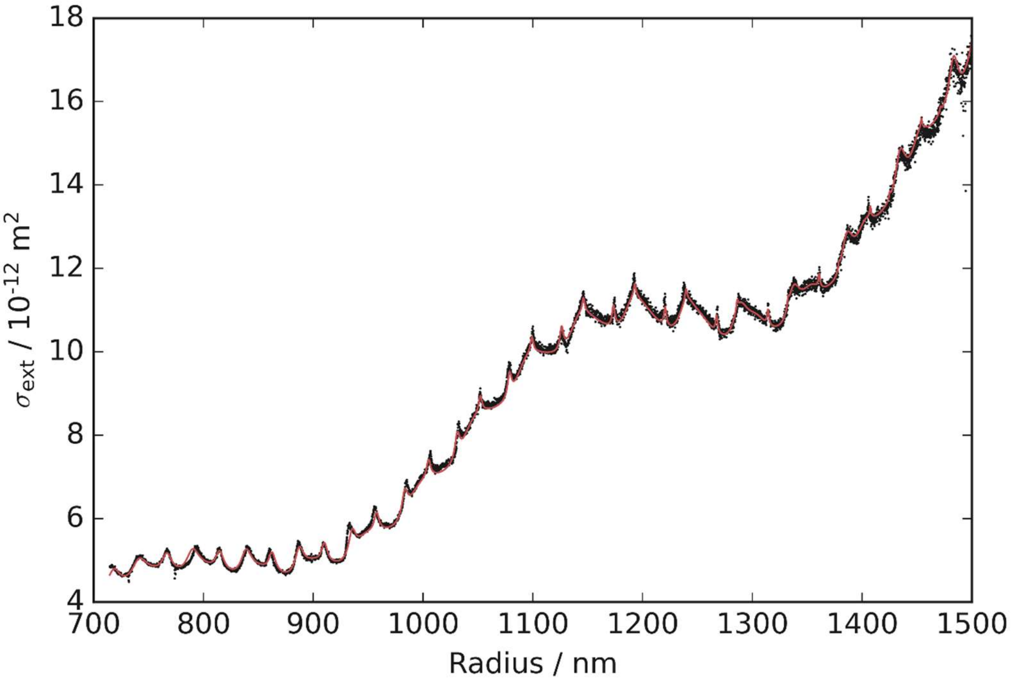

Figure S9. Measured (black points) variation in σ_{ext} with particle r for droplet 2 of 3 for the w_N = 0.002 droplets. The best-fit Mie theory distribution is overlaid (red line). The initial particle radius after complete evaporation of water was 1627 nm. This plot corresponds to the data plotted in Figure 2 of the main manuscript, for w_N = 0.002.

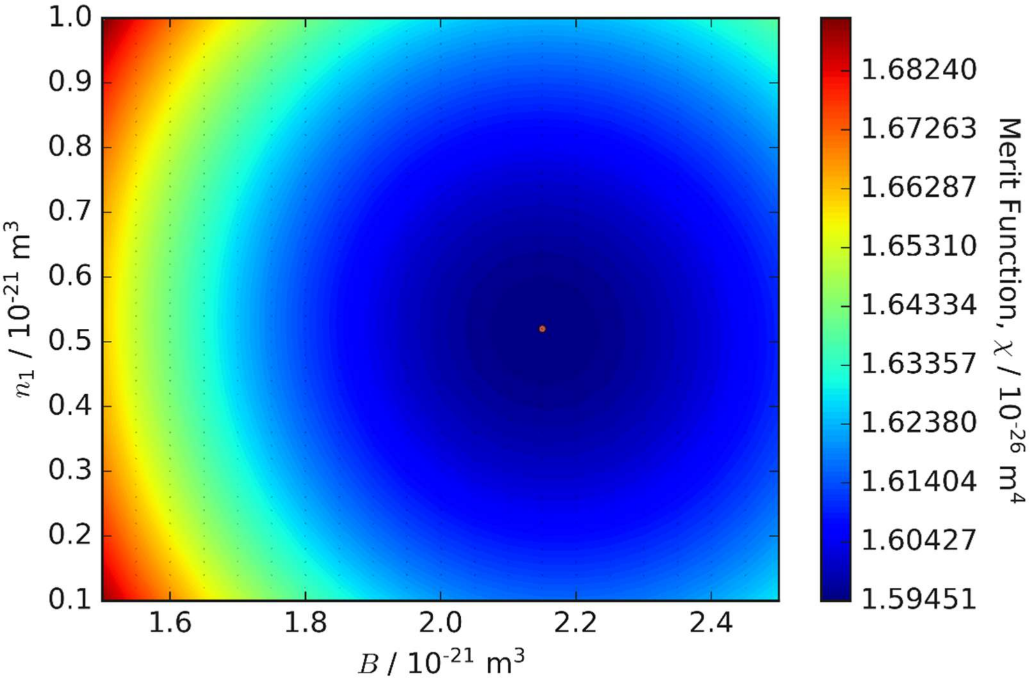

Figure S10. Contour plot depicting the calculated merit functions for droplet 2 of 3 for the w_N = 0.002 droplets, for optimized values of w_0 and the radius scale factor of 255.20 μm and 1.00177, respectively.

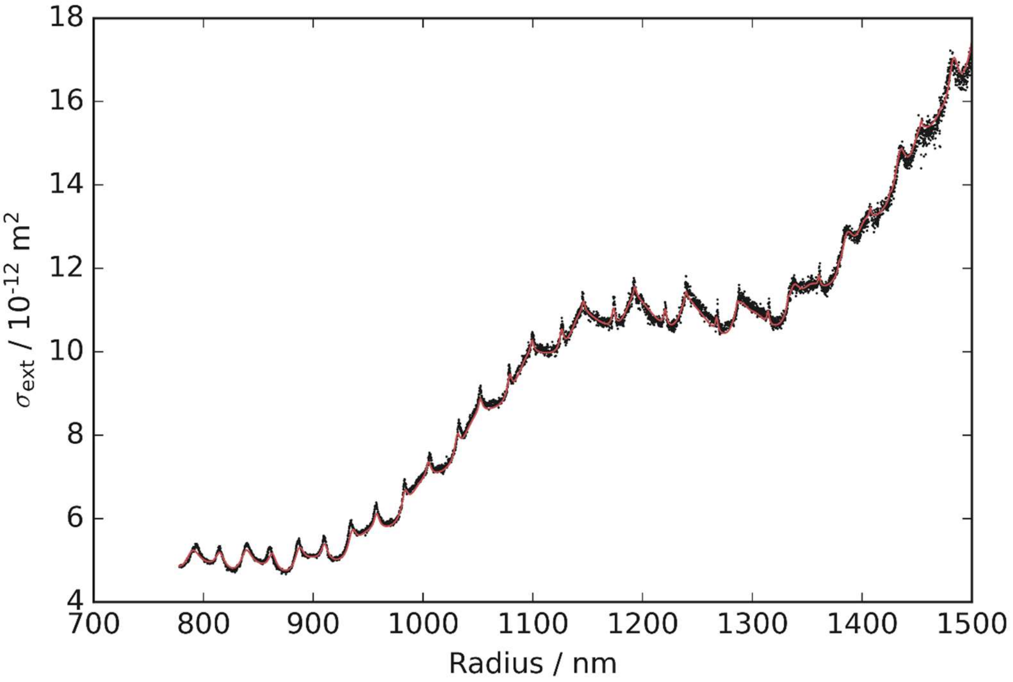

Figure S11. Measured (black points) variation in σ_{ext} with particle r for droplet 3 of 3 for the w_N = 0.002 droplets. The best-fit Mie theory distribution is overlaid (red line). The initial particle radius after complete evaporation of water was 1653 nm.

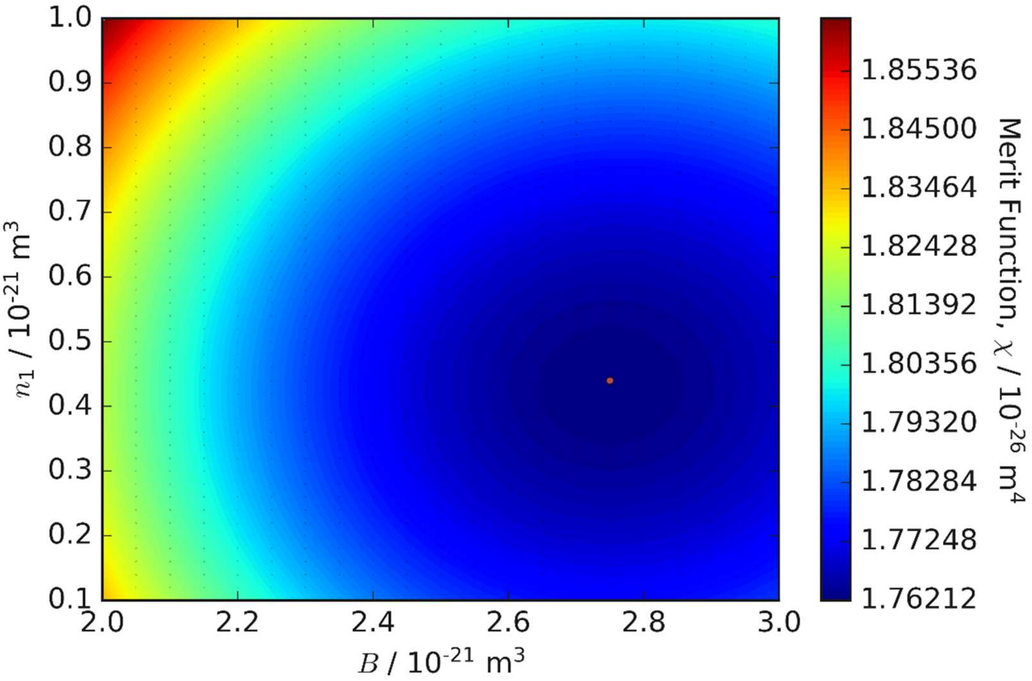

Figure S12. Contour plot depicting the calculated merit functions for droplet 3 of 3 for the w_N = 0.002 droplets, for optimized values of w_0 and the radius scale factor of 257.37 µm and 1.00177, respectively.

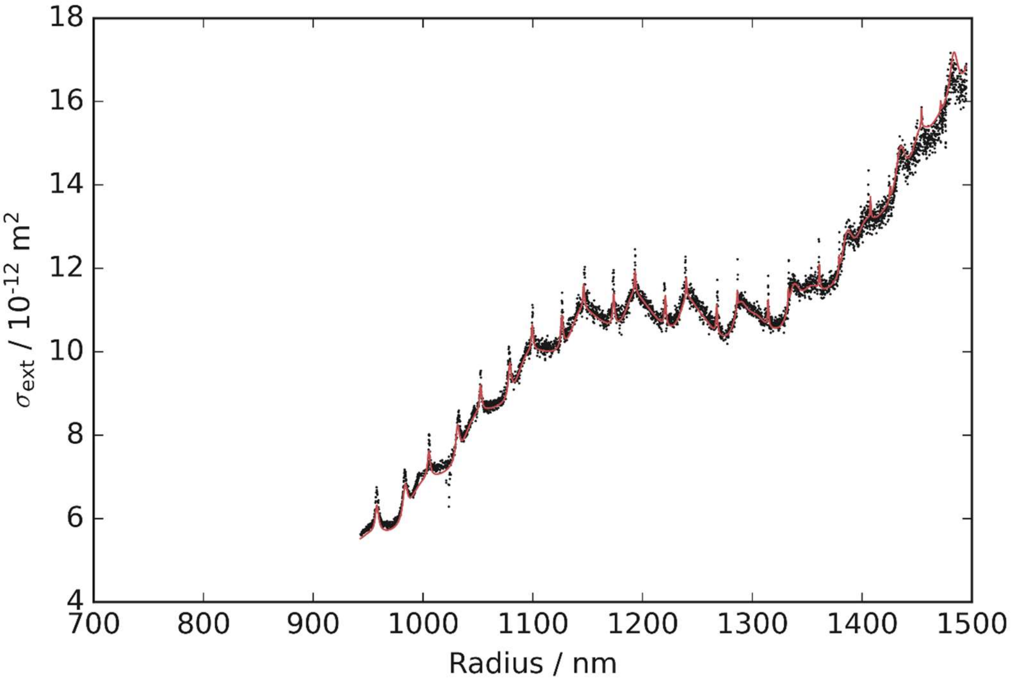

Figure S13. Measured (black points) variation in σ_{ext} with particle r for droplet 1 of 3 for the w_N = 0.000 droplets. The best-fit Mie theory distribution is overlaid (red line). This plot corresponds to the data plotted in Figure 2 of the main manuscript, for w_N = 0.000.

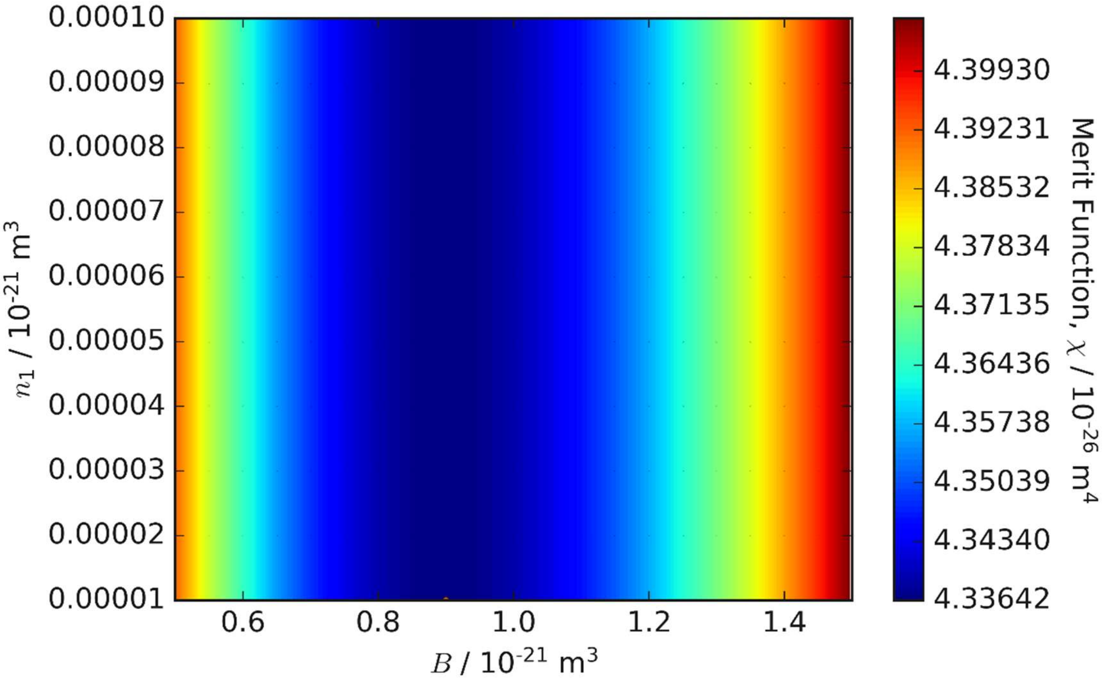

Figure S14. Contour plot depicting the calculated merit functions for droplet 1 of 3 for the w_N = 0.000 droplets, for optimized values of w_0 and the radius scale factor of 260.93 µm and 0.99648, respectively.

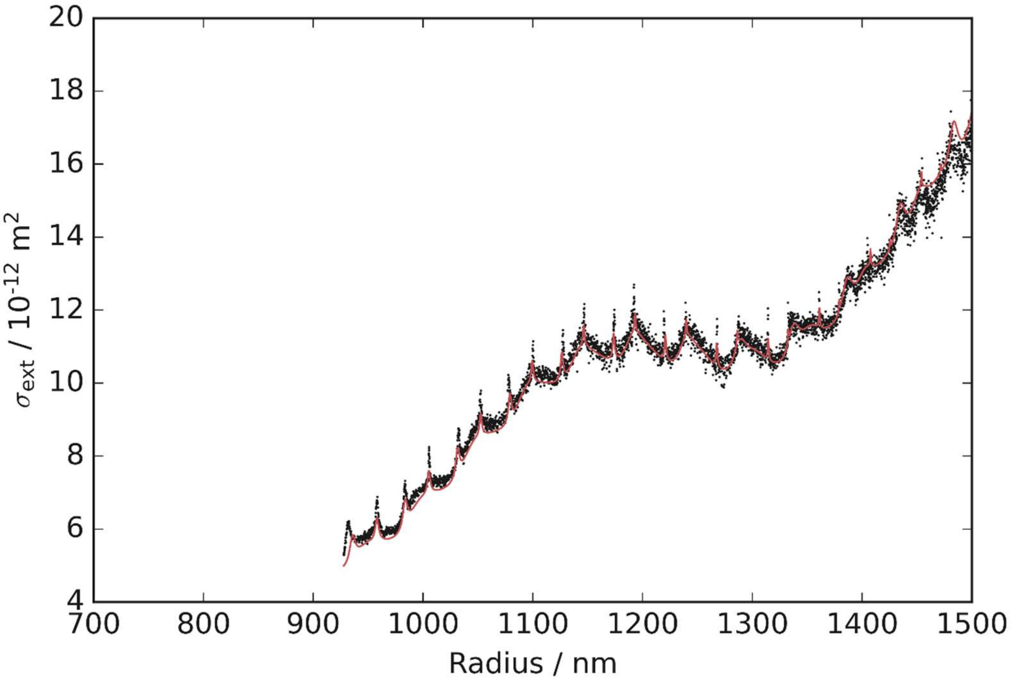

Figure S15. Measured (black points) variation in σ_{ext} with particle r for droplet 2 of 3 for the w_N = 0.000 droplets. The best-fit Mie theory distribution is overlaid (red line).

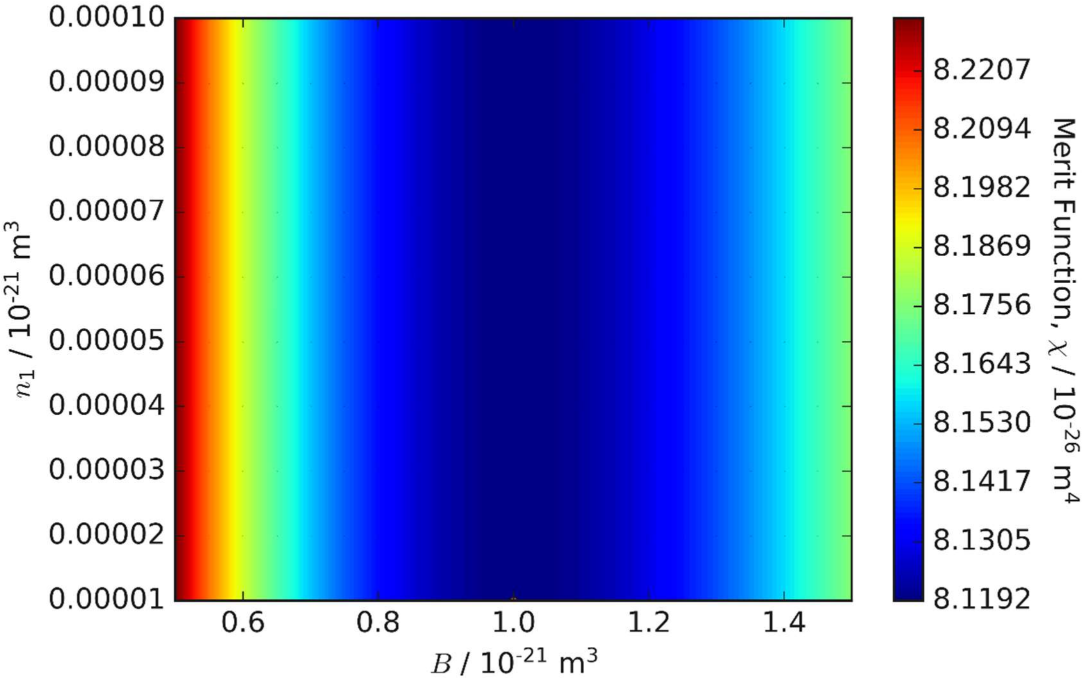

Figure S16. Contour plot depicting the calculated merit functions for droplet 2 of 3 for the w_N = 0.000 droplets, for optimized values of w_0 and the radius scale factor of 263.02 μm and 0.99846, respectively.

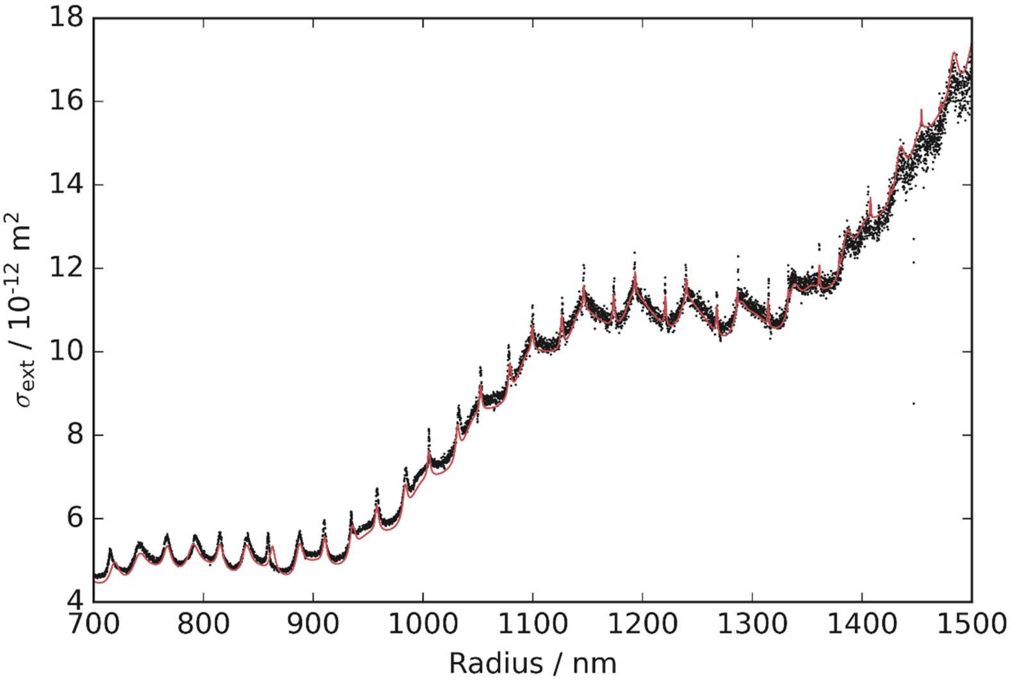

Figure S17. Measured (black points) variation in σ_{ext} with particle r for droplet 3 of 3 for the w_N = 0.000 droplets. The best-fit Mie theory distribution is overlaid (red line).

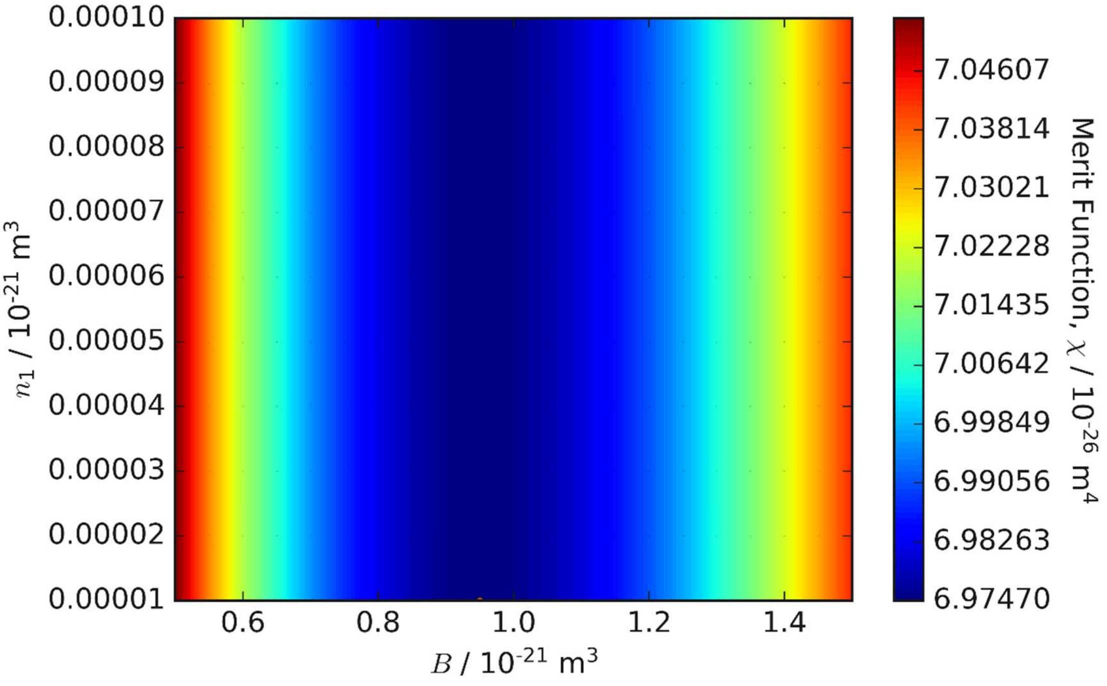

Figure S18. Contour plot depicting the calculated merit functions for droplet 3 of 3 for the w_N = 0.000 droplets, for optimized values of w_0 and the radius scale factor of 260.38 μm and 0.99820, respectively.

References

1. Cotterell, M. I.; Mason, B. J.; Preston, T. C.; Orr-Ewing, A. J.; Reid, J. P., Optical extinction efficiency measurements on fine and accumulation mode aerosol using single particle cavity ring-down spectroscopy. *Phys. Chem. Chem. Phys.* **2015,** *17*, 15843-15856.
2. Liu, Y.; Daum, P. H., Relationship of refractive index to mass density and self-consistency of mixing rules for multicomponent mixtures like ambient aerosols. *J. Aerosol Sci.* **2008,** *39*, 974-986.
3. Cotterell, M. I.; Szpek, K.; Haywood, J. M.; Langridge, J. M., Sensitivity and accuracy of refractive index retrievals from measured extinction and absorption cross sections for mobility-selected internally mixed light absorbing aerosols. *Aerosol Sci. Technol.* **2020,** *54*, 1034-1057.
4. Hasenkopf, C. A.; Beaver, M. R.; Trainer, M. G.; Langley Dewitt, H.; Freedman, M. A.; Toon, O. B.; McKay, C. P.; Tolbert, M. A., Optical properties of Titan and early Earth haze laboratory analogs in the mid-visible. *Icarus* **2010,** *207*, 903-913.
5. Lack, D. A.; Lovejoy, E. R.; Baynard, T.; Pettersson, A.; Ravishankara, A. R., Aerosol Absorption Measurement using Photoacoustic Spectroscopy: Sensitivity, Calibration, and Uncertainty Developments. *Aerosol Sci. Technol.* **2006,** *40*, 697-708.
6. Grineva, O. V.; Zhuravlev, V. I.; Lifanova, N. V., Densities and Dielectric Permittivities of Four Polyhydric Alcohols. *J. Chem. Eng. Data* **1996,** *41*, 155-157.
7. Bond, T. C.; Anderson, T. L.; Campbell, D., Calibration and Intercomparison of Filter-Based Measurements of Visible Light Absorption by Aerosols. *Aerosol Sci. Technol.* **1999,** *30*, 582-600.
8. Sedlacek, A.; Lee, J., Photothermal Interferometric Aerosol Absorption Spectrometry. *Aerosol Sci. Technol.* **2007,** *41*, 1089-1101.
9. Wiegand, J. R.; Mathews, L. D.; Smith, G. D., A UV–Vis Photoacoustic Spectrophotometer. *Anal. Chem.* **2014,** *86*, 6049-6056.
